# Association between Dietary Behavior and Overweight and Obesity among Chinese Students: A Cross-Sectional Study

**DOI:** 10.3390/children10101617

**Published:** 2023-09-28

**Authors:** Jia Hong, Qinghai Gong, Hua Gao, Jinghui Wang, Yanbo Guo, Danjie Jiang, Yan Zhang

**Affiliations:** Ningbo Municipal Center for Disease Control and Prevention, Yongfeng Road 237, Haishu District, Ningbo 315010, China; hongjia1002@163.com (J.H.); gongqinghai@163.com (Q.G.); gailsky@163.com (H.G.); jhnb200210@163.com (J.W.); guoyanbo1983@163.com (Y.G.); jdj0526@yeah.net (D.J.)

**Keywords:** overweight, obesity, dietary behavior, children, students

## Abstract

Objectives: To investigate the prevalence of overweight and obesity among Ningbo students and explore the association between students’ dietary behavior and overweight and obesity. Materials and Methods: A total of 7299 students were recruited, including 3755 males and 3544 females. A questionnaire on student health status and influencing factors was used to investigate dietary behavior. Logistic regression analysis investigated the relationship between dietary behavior and overweight and obesity. Age, gender, area, grade, sleep duration, and physical activity were adjusted in the multivariate regression models. Results: This study revealed that the prevalence of overweight and obesity in Ningbo students was 16.14% and 9.88%, respectively. The logistic regression analysis results showed that regular consumption of sugary beverages was associated with a higher risk of being overweight (OR = 1.256, 95% CI: 1.023–1.542, *p* = 0.029). The research indicated that skipping breakfast was considered a risk factor for obesity (OR = 2.102, 95% CI: 1.087–4.065, *p* = 0.027). After adjusting for age, gender, area, and grade and continuing to adjust for sleep duration and physical activity, the results showed that consuming fried food at least once a day increased the risk of obesity (OR = 1.494, 95% CI: 1.050–2.125, *p* = 0.026; OR = 1.516, 95% CI: 1.065–2.158, *p* = 0.021, respectively). This study found that the frequency of breakfast and the consumption of fried food, fresh vegetables, and fruits were not significantly associated with being overweight (*p* > 0.05). Conclusions: This study indicated that dietary behavior was related to overweight and obesity among Ningbo students. Further studies and more government support are required to confirm this study’s findings and address the current overweight/obesity problems.

## 1. Introduction

Obesity is a complex, multifactorial, noncommunicable disease defined by excessive adiposity [1]. Over the past few decades, the global obesity problem has become increasingly prominent due to rising economic levels, and the number of overweight (OW) and obesity (OB) individuals has increased [2,3]. A study found that global childhood obesity has skyrocketed, with a more than eightfold increase over 40 years [4]. According to a report on nutrition and chronic diseases in Chinese residents (2020) [5], the prevalence of children and adolescents aged 6–17 years with OW and OB reached 11.7% and 7.9%, respectively. Children and adolescents with OW/OB not only affect their growth, development, and mental health [6,7], but they also may have an increased risk of chronic diseases in adulthood, such as hypertension [8,9], diabetes [10,11], and cardiovascular disease [12,13]. Multiple studies have shown that OW/OB in children is independently associated with the risk of cardiovascular disease, cardiovascular disease, and all-cause mortality [14,15], resulting in enormous overall disease costs, including healthcare costs and potential economic losses [16]. Childhood OW/OB has brought significant challenges to children, parents, and even the country. The increased annual total medical costs were USD 237.55 per capita, attributable to childhood OW and OB. OW and OB caused a per capita increase of USD 56.52, USD 14.27, USD 46.38, and USD 1975.06 for costs in nonhospital healthcare, outpatient visits, medication, and hospitalization, respectively. Annual direct and indirect costs were projected to be USD 13.62 billion and USD 49.02 billion by 2050 [17].

With the increasing burden of childhood OW/OB, there is an urgent need for early identification and control of its occurrence and development, which is the key to prevention and control. OW/OB results from a combination of several factors, including genetics [18], environment [19], and behavior [20], among which unhealthy eating habits and patterns are major contributors to increasing OW/OB. For example, research showed that overconsumption of high-fat/sugar-containing foods and beverages contributes to the development of obesity [21]. Different from the influence of genetic factors on OW/OB, external factors such as healthy behavior and lifestyle are controllable, and compared with drug treatment, a change in behavior and lifestyle has a better cost–benefit, which is the most simple, economical, and effective way for children to obtain health [22]. Therefore, the occurrence and development of OW/OB can be prevented and controlled in the early stages by exploring the most beneficial exposure in the behavioral lifestyle to change the adverse behavioral lifestyle. As mentioned above, given the increasing rates of childhood OW/OB and the annual growth in healthcare spending, a study on the association between OW/OB and dietary behavior in students is merited.

Our study was based on the National Monitoring and Intervention Project on Common Diseases and Health Influencing Factors among Students and explored the association between students’ diet and OW/OB. This study aimed to provide a scientific basis for dietary habit-related interventions to prevent OW/OB in schoolchildren.

## 2. Methods

### 2.1. Study Participants

This study was a population-based and cross-sectional study based in Ningbo, Zhejiang, China, from 2019 to 2021, using multistage stratified cluster random sampling. One urban and one rural area were randomly selected in 2019 and 2020, respectively. Two urban areas and one county were selected in 2021. Five schools (two middle schools, two high schools, and one vocational high school) from the urban area and three (two middle schools and two high schools) from the rural area were randomly selected. Then, classes were randomly selected in the school, and investigations were conducted on the whole class, with at least 80 students selected from each grade. A total of 7299 students were enrolled in our study. All participants’ parents or legally authorized representatives signed informed consent forms, and the Ethics Committee of Ningbo Centers for Disease Control and Prevention approved this study (Approval Code: Y2021006, Approval Date: 23 July 2021).

### 2.2. Dietary Behaviour

The survey was based on the National Monitoring and Intervention Project on Common Diseases and Health Influencing Factors among Students. A self-reported questionnaire estimated the students’ dietary behavior. The School Health Center of China Center for Disease Control and Prevention drafted, validated, and distributed the questionnaire. The questionnaire investigation was conducted with the class as a whole by professionally trained investigators, including the students’ basic information (gender, age, area, and grade); eating habits (frequency of sweetened beverages, fried food, fresh fruits, and vegetable consumption); and life behavior (frequency of breakfast eating, physical activity, and sleep duration). The consumption of sweetened beverages, fried food, fresh fruits, and vegetables was identified by asking the students, “How many times have you consumed sweetened beverages/fried food/fresh fruits/vegetables in the past seven days?”. The frequency of breakfast eating was identified by asking the students “Do you have breakfast every day in the past seven days?”.

### 2.3. Overweight and Obesity

Students’ physical examinations were scheduled on an empty stomach in the morning. In the daily examination, the retested objects were randomly selected in a proportion of 5%. The original examiner retested the physical examinations to test the error, and the allowable range of the test error was 0.5 cm for height and 0.1 kg for weight. OW/OB was distinguished according to the “Body mass index reference norm for screening overweight and obesity in Chinese children and adolescents” [23,24].

### 2.4. Statistical Analysis

The data of this study were established using EpiData 3.1 and analyzed by SPSS 19.0. The characteristics of the study subjects were described by the mean and standard deviation for continuous variables and by numbers and percentages for categorical variables. Logistic regression models were used in this study to investigate the association of the frequency of breakfast eating (treated as a tri-categorical variable: “every morning”, “only on occasion”, or “never”), the consumption of sweetened beverages (treated as a tri-categorical variable: “never”, “less than once a day”, or “once a day or more”), fried food (treated as a tri-categorical variable: “never”, “less than once a day”, or “once a day or more”), fruit (treated as a quad-categorical variable: “never”, “less than once a day”, “once a day or more”, or “two or more times a day”), and vegetables (treated as a quad-categorical variable: “never”, “less than once a day”, “once a day or more”, “two or more times a day”, or “two or more times a day”) with OW/OB. The odds ratios (ORs) and 95% confidence intervals (95% CIs) were used to assess the risk of OW/OB. Multivariable-adjusted Model 1 was adjusted for age, sex, area, and grade, and Model 2 was further adjusted for sleep duration and physical activity based on Model 1. GraphPad Prism 8 was used to draw the forest plot. A *p* value of <0.05 was considered statistically significant.

## 3. Results

### 3.1. General Characteristics

The general characteristics of the study participants are shown in Table 1. Among the 7299 students included, 51.45% were male, 66.68% were living in urban areas, 48.38% were junior middle school students, 39.28% were senior middle school students, 12.34% were vocational middle school students, and the average age was 15.09 ± 1.81 years.

There were 1178 students with OW and 721 students with OB, and the OW and OB rates were 16.14% and 9.88%, respectively. Students with OW/OB were younger (*p* < 0.001), more likely to be males (*p* < 0.001), junior middle school students (*p* < 0.001), and had physical activity between 1 and 2 h a day (*p* = 0.040).

### 3.2. Dietary Factors and Overweight

**Figure 1:**
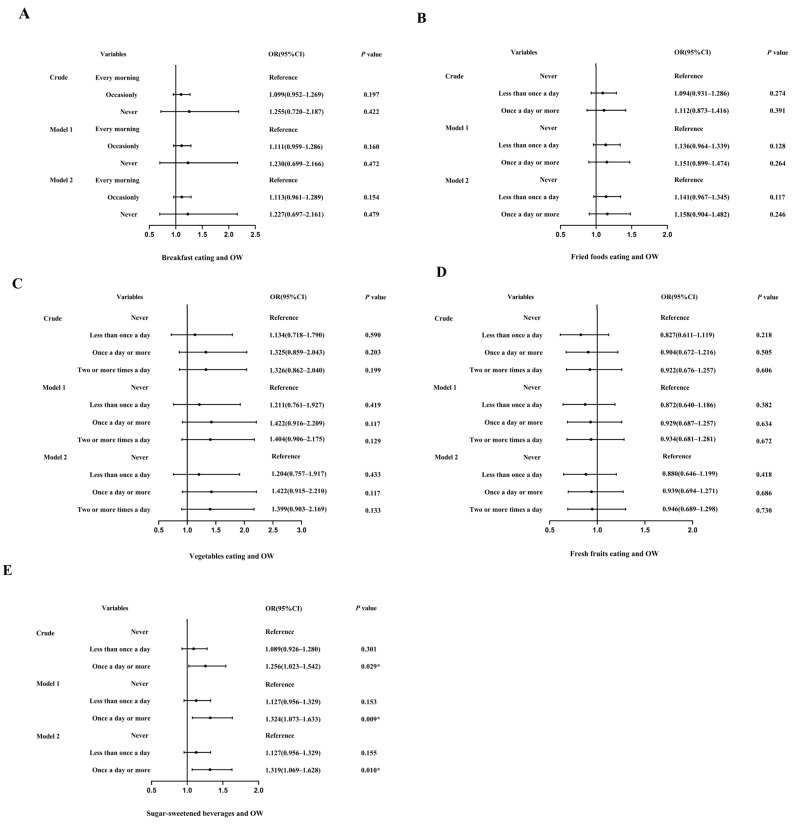
Logistic regression analysis of overweight and dietary behavior. (**A**). Logistic regression analysis of overweight and breakfast eating. (**B**). Logistic regression analysis of overweight and fried food eating. (**C**). Logistic regression analysis of overweight and vegetable eating. (**D**). Logistic regression analysis of overweight and fresh fruit eating. (**E**). Logistic regression analysis of overweight and sugar-sweetened beverages. Notes: OW, overweight; OR, odds ratio; CI, confidence interval; crude: confounding factors were not adjusted; Model 1, adjusted for age, gender, area, and grade; Model 2, sleep duration and physical activity were further adjusted based on Model 1. *, *p* < 0.05. The vertical line in the figure was an invalid line (OR = 1), the small dots were ORs, and the horizontal line represented the 95% CI.

Figure 1 presents ORs and 95% CIs for the association between dietary factors and OW. There was no significant difference in the OW rate between the occasional breakfast eater (*p* = 0.197), breakfast skipper (*p* = 0.422), and regular breakfast eater (Figure 1A). Similarly, in the consumption of fried foods (Figure 1B), fresh vegetables (Figure 1C), and fruits (Figure 1D), it was observed that the frequency of consumption was not related to OW (*p* > 0.05). Then, we studied the association between the frequency of consumption of sugary beverages and OW (Figure 1E). The results from the crude and multivariable-adjusted models indicated no statistically significant difference in the risk of OW between occasional drinkers and nondrinkers (*p* > 0.05). However, regular drinkers were at a higher risk of OW than nondrinkers (OR = 1.256, 95% CI: 1.023–1.542, *p* = 0.029), which remained statistically significant after adjusting for age, sex, area, and grade (Model 1: OR = 1.324, 95% CI: 1.073–1.633, *p* = 0.009) and further adjusting for sleep duration and physical activity (Model 2: OR = 1.319, 95% CI: 1.069–1.628, *p* = 0.010).

### 3.3. Dietary Factors and Obesity

**Figure 2:**
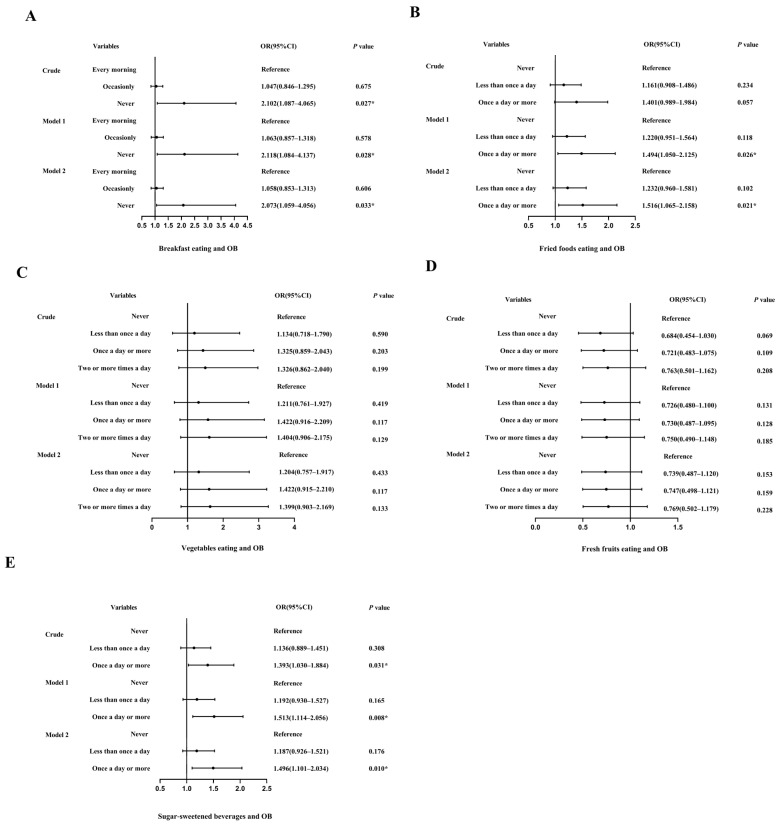
Logistic regression analysis of obesity and dietary behavior. (**A**). Logistic regression analysis of obesity and breakfast eating. (**B**). Logistic regression analysis of obesity and fried food eating. (**C**). Logistic regression analysis of obesity and vegetable eating; (**D**). Logistic regression analysis of obesity and fresh fruit eating. (**E**). Logistic regression analysis of obesity and sugar-sweetened beverages. Notes: OB, obesity; OR, odds ratio; CI, confidence interval; crude: confounding factors were not adjusted. Model 1, adjusted for age, gender, area, and grade; Model 2, sleep duration, and physical activity were further adjusted based on Model 1. *, *p* < 0.05. The vertical line in the figure was an invalid line (OR = 1), the small dots were ORs, and the horizontal line represented the 95% CI.

In Figure 2A, the research results showed no difference in the risk of OB between students who eat breakfast regularly every day and those who occasionally eat breakfast (*p* = 0.675). However, the OB rate of breakfast skippers was significantly higher than those of regular breakfast eaters (OR = 2.102, 95% CI: 1.087–4.065, *p* = 0.027). In multivariable-adjusted models, breakfast skippers still reported a higher risk of OB (Model 1: OR = 2.118, 95% CI: 1.084–4.137, *p* = 0.028; Model 2: OR = 2.073, 95% CI: 1.059–4.056, *p* = 0.033, respectively). In the analysis of the association between eating fried food and OB (Figure 2B), after adjusting for age, gender, area, and grade, eating fried foods at least once a day was considered a risk factor for OB (OR = 1.494, 95% CI: 1.050–2.125, *p* = 0.026). After continuing to adjust for sleep timing and exercise (OR = 1.516, 95% CI: 1.065–2.158, *p* = 0.021), the students who ate fried food at least once a day continued to have a higher risk of OB. Then, this study analyzed the frequency of eating vegetables (Figure 2C) and fresh fruits (Figure 2D) and the risk of OB and found no statistical significance in the risk of OB (*p* > 0.05). Finally, in the relationship between the frequency of consumption of sugary beverages and OB (Figure 2E), the results showed no statistically significant difference in the risk of OB between occasional drinkers and nondrinkers (*p* = 0.308). However, regular drinkers were at a higher risk of OB than nondrinkers (OR = 1.393, 95% CI: 1.030–1.884, *p* = 0.031), which remained statistically significant after adjusting for the variables (Model 1: OR = 1.513, 95% CI: 1.114–2.056, *p* = 0.008, Model 2: OR = 1.496, 95% CI: 1.101–2.034, *p* = 0.010, respectively).

## 4. Discussion

The World Health Organization has defined OW/OB as an epidemic [25]. Childhood and adolescence with OW/OB have become significant global public health problems [26]. Studies have predicted that the prevalence of OW/OB among children and adolescents in China could reach 31.8% by 2030, and the medical costs associated with OB could account for 22% of the total national medical expenditure [27]. This survey showed that the prevalence of OW and OB in Ningbo was 26.02%, higher than the 23.4% national detection rate in children and adolescents aged 7–18 years in 2019 [28]. This highlights the severity of childhood and adolescence with OW/OB in Ningbo, and more attention should be given to this issue. Researchers have indicated that environmental and socioeconomic factors play an essential role in the susceptibility and development of OW/OB [29,30]. Studies have found that socioeconomic development, represented by an increase in per capita gross domestic product and urbanization rates and a decrease in the Engel coefficient, is accompanied by improved stunting and wasting but a rapid increase in OW/OB [31,32,33,34]. As one of the leading cities in China, the economic growth of Ningbo has led to an increase in the residents’ household income, supporting the residents to spend more on the daily diet, thus expanding the diversity of the diet and resulting in increased consumption of high-energy foods [35]. Economic growth has provided a basis for the prevalence of OW/OB in children. However, unhealthy lifestyles, such as lack of physical activity [36], shortened sleep duration, and other factors [37,38], contribute more to childhood OW/OB. Our research was based on monitoring data from 2019 to 2020 during the coronavirus infection (COVID-19) pandemic. The higher rate of overweight and obesity compared with the national data for 2019 may also be due to the different living habits and nutritional status before and after COVID-19 [39]. Social distancing and online classes led to students staying home for a long time without going out. With the increase in time at home and screen time, dietary behaviors have also changed, increasing the intake of fast food, snacks, and high-calorie foods [40].

Our study also observed gender differences in the prevalence of OW/OB in students, with a higher proportion of boys with OW/OB than girls. According to Chen et al.’s research in Chongqing, China, the overweight and obesity rates of boys in children (aged 6–12 years) were higher than those of girls (15.97% vs. 10.51%, 12.33% vs. 4.53%, *p* < 0.001), which was consistent with our research [41]. The same results were also observed in studies in Suzhou and Shanghai, China [42,43]. This may be due to the differences in attitudes toward OW/OB in China [44]. Compared with boys, Chinese girls tend to pay more attention to their body shape, prefer slim and lean figures, and are more likely to control their weight [45]. Unlike girls, many parents consider heavier boys physically healthy and strong, leading to greater tolerance for OW/OB in boys. This may be one major reason for the difference in OW/OB rates between male and female students.

Dietary factors are another vital factor affecting OW/OB in children and adolescents [46]. In this study, we also investigated the association between students’ dietary behavior and OW/OB and found that regular consumption of sugary beverages was associated with a higher risk of being OW compared to students who never consumed; this is consistent with the results of other studies [47,48]. The present research also indicated that skipping breakfast was considered a risk factor for OB. The mechanism behind how skipping breakfast leads to OW/OB is still unclear. This may be due to excessive snacking among students who skip breakfast, and the energy intake from the snacks exceeds the energy required from breakfast [49]. Studies have reported that breakfast skippers have significantly decreased protein and dietary fiber intake and have lower physical activity than breakfast eaters, and skipping breakfast increases serum small dense low-density lipoprotein cholesterol concentration and causes unfavorable lipid profiles [50,51]. Research has demonstrated that eating breakfast can improve diet quality and increase physical activity [52]. Although fruits and vegetables are widely recognized as protective foods for childhood OB, weight management programs almost always encourage children and adolescents to eat more [53]. However, this study showed no significant association between the frequency of fruits and vegetables and OW/OB. This may be due to the limitations of the cross-sectional study for this survey. Students with OW/OB deliberately changed their eating behavior, such as increasing the intake of vegetables and fruits, which led to biased findings. Therefore, further research is needed to explore the relationship between the intake of vegetables and fruits and childhood OW/OB.

The strengths of this study were that it included a relatively large number of research subjects from several different schools and years and adjusted for possible confounding factors. However, this study still has certain limitations. First, as this is a cross-sectional study, it is essential to note that the associations observed may be bidirectional, and there is no clear evidence of the results. Second, in this study, dietary factors were estimated using a questionnaire to investigate the frequency of consumption, which may be affected by recall bias. The specific dietary intake such as other sugary foods that play an important role in overweight and obesity, was not considered in this study, which may cause some errors in evaluating the results. Moreover, different definitions of overweight and obesity in children and adolescents have been used in China and other countries, which add complexities to making comparisons across studies [54]. Therefore, more diverse research factors should be studied in future research, and further prospective cohort studies are warranted if permitted.

In summary, the rate of children and adolescents with OW/OB in Ningbo is relatively high despite the government implementing a health education program and issuing dietary guidelines to counter the threat of OW/OB. These measures aim to increase people’s knowledge of nutrition, promote the development of healthy dietary patterns, and maintain a healthy lifestyle. Despite progress in preventing OW/OB in children and adolescents, key factors that must be addressed remain uncertain. Among students, we should pay attention to boys and junior high school students, especially those with frequent consumption of sugary drinks, skipping breakfast, and regular fried food. Health education for children and adolescents should be strengthened, and healthy dietary habits should be developed. At the same time, nutrition knowledge for parents should also be emphasized to control the risk of OW/OB in children and adolescents.

## 5. Conclusions

The prevalence of overweight and obesity among children and adolescents in Ningbo was relatively high, and the frequency of consumption of breakfast, fried foods, and sugary beverages was related to overweight/obesity. Further studies and more government support are required to confirm the findings of this study and address current overweight/obesity problems. Health education should be strengthened for students, parents, and schools to change their dietary habits and prevent and control the occurrence and development of OW/OB.

## Figures and Tables

**Table 1:** General characteristics of subjects in the OW group, OB group, and non-OW/OB group.

Characteristics	Total (*n* = 7299)	Non-OW/OB Group (*n* = 5400)	OW Group (*n* = 1178)	OB Group (*n* = 721)	*p* Value
Prevalence (%)	-	-	16.14	9.88	
Age	15.09 ± 1.81	15.15 ± 1.80	15.05 ± 1.85	14.74 ± 1.79	<0.001
Gender					
Male	3755 (51.45)	2497 (46.24)	754 (64.01)	504 (69.90)	<0.001
Female	3544 (48.55)	2903 (53.76)	424 (35.99)	217 (30.10)	
Area					
Urban	4867 (66.68)	3604 (66.74)	784 (66.55)	479 (66.44)	0.982
Rural	2432 (33.32)	1796 (33.26)	394 (33.45)	242 (33.56)	
Grade					
Junior middle school	3531 (48.38)	2551 (47.24)	570 (48.39)	410 (56.87)	<0.001
Senior middle school	2867 (39.28)	2159 (39.98)	478 (40.58)	230 (31.90)	
Vocational senior middle school	901 (12.34)	690 (12.78)	130 (11.04)	81 (11.23)	
Sleep duration (h/day)	7.70 ± 1.09	7.69 ± 1.09	7.72 ± 1.09	7.75 ± 1.10	0.229
Physical activity/day					
<1 h	1734 (23.76)	1328 (24.59)	254 (21.56)	152 (21.08)	0.040
1–2 h	2872 (39.35)	2110 (39.07)	485 (41.17)	277 (38.42)	
2–3 h	1104 (15.13)	800 (14.81)	179 (15.20)	125 (17.34)	
≥3 h	1287 (17.63)	946 (17.52)	216 (18.34)	125 (17.34)	
Unknown	302 (4.14)	216 (4.00)	44 (3.74)	42 (5.83)	
Frequency of breakfast eating					
Every morning	6125 (83.92)	4549 (84.24)	975 (82.77)	601 (83.36)	0.281
Only on occasion	1114 (15.26)	809 (14.98)	195 (16.55)	110 (15.26)	
Never	60 (0.82)	42 (0.78)	8 (0.68)	10 (1.39)	
Frequency of sugary drinks eating					
Never	941 (12.89)	715 (13.24)	145 (12.31)	81 (11.23)	0.168
Less than once a day	5401 (74.00)	4001 (74.09)	871 (73.94)	529 (73.37)	
Once a day or more	957 (13.11)	684 (12.67)	162 (13.75)	111 (15.40)	
Frequency of fried food eating					
Never	923 (12.65)	699 (12.94)	145 (12.31)	79 (10.96)	0.318
Less than once a day	5824 (79.79)	4294 (79.52)	952 (80.81)	578 (80.17)	
Once a day or more	552 (7.56)	407 (7.54)	81 (6.88)	64 (8.88)	
Frequency of fresh fruit eating					
Never	231 (3.16)	166 (3.07)	36 (3.06)	29 (4.02)	0.425
Less than once a day	2192 (30.03)	1654 (30.63)	337 (28.61)	201 (27.88)	
Once a day or more	3458 (47.38)	2544 (47.11)	574 (48.73)	340 (47.16)	
Two or more times a day	1418 (19.43)	1036 (19.19)	231 (19.61)	151 (20.94)	
Frequency of vegetables eating					
Never	128 (1.75)	101 (1.87)	18 (1.53)	9 (1.25)	0.377
Less than once a day	761 (10.43)	584 (10.81)	114 (9.68)	63 (8.74)	
Once a day or more	2688 (36.83)	1977 (36.61)	446 (37.86)	265 (36.75)	
Two or more times a day	3722 (50.99)	2738 (50.70)	600 (50.93)	384 (53.26)	
BMI (kg/m^2^)	20.96 ± 3.86	19.19 ± 2.07	24.26 ± 1.53	28.82 ± 3.47	-

OW, overweight; OB, obesity; BMI, body mass index.

## Data Availability

The datasets analyzed during the current study are available from the corresponding author on reasonable request.

## References

[1] World Health Organization (2021). Draft Recommendations for the Prevention and Management of Obesity over the Life Course, Including Potential Targets.

[2] Li C., Zhang M., Tarken A.Y., Cao Y., Li Q., Wang H. (2023). Secular trends and sociodemographic determinants of thinness, overweight and obesity among Chinese children and adolescents aged 7–18 years from 2010 to 2018. Front. Public Health.

[3] Afshin A., Forouzanfar M.H., Reitsma M.B., Sur P., Estep K., Lee A., Marczak L., Mokdad A.H., Moradi-Lakeh M., Naghavi M. (2017). Health Effects of Overweight and Obesity in 195 Countries over 25 Years. N. Engl. J. Med..

[4] NCD Risk Factor Collaboration (NCD-RisC) (2017). Worldwide trends in body-mass index, underweight, overweight, and obesity from 1975 to 2016: A pooled analysis of 2416 population-based measurement studies in 128·9 million children, adolescents, and adults. Lancet.

[5] The State Council Information Office of the People’s Republic of China Press Conference on the “Report on the Status of Nutrition and Chronic Diseases of Chinese Residents (2020)”. https://www.gov.cn/xinwen/2020-12/24/content_5572983.htm.

[6] Frank D.M., Bradshaw P.T., Mujahid M., Epel E., Lararia B.A. (2023). Adolescent BMI trajectory and associations with adult metabolic syndrome and offspring obesity. Obesity.

[7] Hagman E., Danielsson P., Brandt L., Ekbom A., Marcus C. (2016). Association between impaired fasting glycaemia in pediatric obesity and type 2 diabetes in young adulthood. Nutr. Diabetes.

[8] Koebnick C., Sidell M.A., Li X., Woolford S.J., Kuizon B.D., Kunani P. (2023). Association of High Normal Body Weight in Youths with Risk of Hypertension. JAMA Netw. Open.

[9] Li C., Liu Z., Zhao M., Zhang C., Bovet P., Xi B. (2023). Weight status change from birth to childhood and the odds of high blood pressure among Chinese children. Front. Public Health.

[10] Huang Y., Xu Y., Qiao Y., Wang H., Zhong V.W. (2023). Quantifying the contribution of 31 risk factors to the increasing prevalence of diabetes among US adults, 2005–2018. Front. Public Health.

[11] Lindberg L., Danielsson P., Persson M., Marcus C., Hagman E. (2020). Association of childhood obesity with risk of early all-cause and cause-specific mortality: A Swedish prospective cohort study. PLoS Med..

[12] Sommer A., Twig G. (2018). The Impact of Childhood and Adolescent Obesity on Cardiovascular Risk in Adulthood: A Systematic Review. Curr. Diabetes Rep..

[13] Weihrauch-Blüher S., Wiegand S. (2018). Risk Factors and Implications of Childhood Obesity. Curr. Obes. Rep..

[14] Blüher M. (2019). Obesity: Global epidemiology and pathogenesis. Nat. Rev. Endocrinol..

[15] Smith J.D., Fu E., Kobayashi M.A. (2020). Prevention and Management of Childhood Obesity and Its Psychological and Health Comorbidities. Annu. Rev. Clin. Psychol..

[16] Ng M., Fleming T., Robinson M., Thomson B., Graetz N., Margono C., Mullany E.C., Biryukov S., Abbafati C., Abera S.F. (2014). Global, regional, and national prevalence of overweight and obesity in children and adults during 1980-2013: A systematic analysis for the Global Burden of Disease Study 2013. Lancet.

[17] Ling J., Chen S., Zahry N.R., Kao T.-S.A. (2023). Economic burden of childhood overweight and obesity: A systematic review and meta-analysis. Obes. Rev. Off. J. Int. Assoc. Study Obes..

[18] Willyard C. (2014). Heritability: The family roots of obesity. Nature.

[19] Albuquerque D., Nóbrega C., Manco L., Padez C. (2017). The contribution of genetics and environment to obesity. Br. Med. Bull..

[20] Ishida Y., Yoshida D., Honda T., Hirakawa Y., Shibata M., Sakata S., Furuta Y., Oishi E., Hata J., Kitazono T. (2020). Influence of the Accumulation of Unhealthy Eating Habits on Obesity in a General Japanese Population: The Hisayama Study. Nutrients.

[21] Jacques A., Chaaya N., Beecher K., Ali S.A., Belmer A., Bartlett S. (2019). The impact of sugar consumption on stress driven, emotional and addictive behaviors. Neurosci. Biobehav. Rev..

[22] Dabas A., Seth A. (2018). Prevention and Management of Childhood Obesity. Indian J. Pediatr..

[23] Group of China Obesity Task Force (2004). Body mass index reference norm for screening overweight and obesity in Chinese children and adolescents. Zhonghua Liu Xing Bing Xue Za Zhi.

[24] Ji C.-Y. (2005). Report on childhood obesity in China (1)—Body mass index reference for screening overweight and obesity in Chinese school-age children. Biomed Environ. Sci..

[25] James W.P.T. (2008). WHO recognition of the global obesity epidemic. Int. J. Obes..

[26] Di Cesare M., Sorić M., Bovet P., Miranda J.J., Bhutta Z., Stevens G.A., Laxmaiah A., Kengne A.P., Bentham J. (2019). The epidemiological burden of obesity in childhood: A worldwide epidemic requiring urgent action. BMC Med..

[27] Gao L., Wu Y., Chen S., Zhou H., Zhao L., Wang Y. (2022). Time trends and disparities in combined overweight and obesity prevalence among children in China. Nutr. Bull..

[28] Dong Y.H., Chen L., Liu J.Y., Ma T., Zhang Y., Chen M.M., Zhong P.L., Shi D., Hu P.J., Li J. (2023). Epidemiology and prediction of overweight and obesity among children and adolescents aged 7–18 years in China from 1985 to 2019. Zhonghua Yu Fang Yi Xue Za Zhi.

[29] Zhao J., Fan B., Huang J., Cowling B.J., Au Yeung S.L.R., Baccarelli A., Leung G.M., Schooling C.M. (2023). Environment- and epigenome-wide association study of obesity in ‘Children of 1997’ birth cohort. eLife.

[30] Anekwe C.V., Jarrell A.R., Townsend M.J., Gaudier G.I., Hiserodt J.M., Stanford F.C. (2020). Socioeconomics of Obesity. Curr. Obes. Rep..

[31] Song Y., Wang H.J., Dong B., Ma J., Wang Z., Agardh A. (2016). 25-year trends in gender disparity for obesity and overweight by using WHO and IOTF definitions among Chinese school-aged children: A multiple cross-sectional study. BMJ Open.

[32] You W., Henneberg M. (2022). Significantly different roles of economic affluence in sex-specific obesity prevalence rates: Understanding more modifications within female body weight management. Sci. Rep..

[33] Dumith S.C., Saes-Silva E., Languer Vargas B., Belarmino V., Volz P.M., Nascimento da Silva C., de Oliveira Meller F., Schäfer A.A., Pereira da Silva M. (2022). What factors explain the increase in obesity in Brazil? An ecological analysis of contextual and behavioural components. Public Health.

[34] Dong Y., Jan C., Ma Y., Dong B., Zou Z., Yang Y., Xu R., Song Y., Ma J., Sawyer S.M. (2019). Economic development and the nutritional status of Chinese school-aged children and adolescents from 1995 to 2014: An analysis of five successive national surveys. Lancet Diabetes Endocrinol..

[35] Zhang Y., Lou H., Huang Y., Wang R., Wen X., Wu C., Hao C., Li R., Gao G., Lou X. (2022). Trends of overweight and obesity prevalence in school-aged children among Henan Province from 2000 to 2019. Front. Public Health.

[36] Hills A.P., Andersen L.B., Byrne N.M. (2011). Physical activity and obesity in children. Br. J. Sports Med..

[37] Fan J., Ding C., Gong W., Yuan F., Zhang Y., Feng G., Song C., Liu A. (2020). Association of Sleep Duration and Overweight/Obesity among Children in China. Int. J. Environ. Res. Public Health.

[38] Tambalis K.D., Panagiotakos D.B., Psarra G., Sidossis L.S. (2018). Insufficient Sleep Duration Is Associated with Dietary Habits, Screen Time, and Obesity in Children. J. Clin. Sleep Med. JCSM Off. Publ. Am. Acad. Sleep Med..

[39] Choi J.E., Lee H.A., Park S.W., Lee J.W., Lee J.H., Park H., Kim H.S. (2023). Increase of Prevalence of Obesity and Metabolic Syndrome in Children and Adolescents in Korea during the COVID-19 Pandemic: A Cross-Sectional Study Using the KNHANES. Children.

[40] Sanyaolu A., Okorie C., Marinkovic A., Patidar R., Younis K., Desai P., Hosein Z., Padda I., Mangat J., Altaf M. (2020). Comorbidity and its Impact on Patients with COVID-19. SN Compr. Clin. Med..

[41] Chen J., Luo S., Liang X., Luo Y., Li R. (2021). The relationship between socioeconomic status and childhood overweight/obesity is linked through paternal obesity and dietary intake: A cross-sectional study in Chongqing, China. Environ. Health Prev. Med..

[42] Hu J., Ding Z., Han D., Hai B., Lv H., Yin J., Shen H., Gu A., Yang H. (2022). Prevalence of hypertension and related risk factors among children and adolescents at three separate visits: A large school-based study in China. Front. Pediatr..

[43] Liu Y., Sun X., Zhang E., Li H., Ge X., Hu F., Cai Y., Xiang M. (2023). Association between Types of Screen Time and Weight Status during the COVID-19 Pandemic: A Longitudinal Study in Children and Adolescents. Nutrients.

[44] Li B., Adab P., Cheng K.K. (2015). The role of grandparents in childhood obesity in China—Evidence from a mixed methods study. Int. J. Behav. Nutr. Phys. Act..

[45] Song Y., Wang H.J., Ma J., Wang Z. (2013). Secular trends of obesity prevalence in urban Chinese children from 1985 to 2010: Gender disparity. PLoS ONE.

[46] Magriplis E., Michas G., Petridi E., Chrousos G.P., Roma E., Benetou V., Cholopoulos N., Micha R., Panagiotakos D., Zampelas A. (2021). Dietary Sugar Intake and Its Association with Obesity in Children and Adolescents. Children.

[47] Sang Iii C.J., de Visser R., Krallman R., Pai C.-W., Montgomery D., Moser C.A., Kline-Rogers E., DuRussel-Weston J., Eagle K.A., Chinapaw M. (2023). Cardiometabolic Risk and Dietary Behaviors in Middle-School Children Consuming School-Sourced Lunch. Acad. Pediatr..

[48] Alsulami S., Althagafi N., Hazazi E., Alsayed R., Alghamdi M., Almohammadi T., Almurashi S., Baig M. (2023). Obesity and Its Associations with Gender, Smoking, Consumption of Sugary Drinks, and Hour of Sleep Among King Abdulaziz University Students in Saudi Arabia. Diabetes Metab. Syndr. Obes..

[49] Dubois L., Girard M., Potvin Kent M., Farmer A., Tatone-Tokuda F. (2009). Breakfast skipping is associated with differences in meal patterns, macronutrient intakes and overweight among pre-school children. Public Health Nutr..

[50] Zeballos E., Todd J.E. (2020). The effects of skipping a meal on daily energy intake and diet quality. Public Health Nutr..

[51] Arimoto M., Yamamoto Y., Imaoka W., Kuroshima T., Toragai R., Nakamura M., Ito Y., Ai M. (2023). Small Dense Low-Density Lipoprotein Cholesterol Levels in Breakfast Skippers and Staple Foods Skippers. J. Atheroscler. Thromb..

[52] Schembre S.M., Wen C.K., Davis J.N., Shen E., Nguyen-Rodriguez S.T., Belcher B.R., Hsu Y.W., Weigensberg M.J., Goran M.I., Spruijt-Metz D. (2013). Eating breakfast more frequently is cross-sectionally associated with greater physical activity and lower levels of adiposity in overweight Latina and African American girls. Am. J. Clin. Nutr..

[53] Folkvord F., Naderer B., Coates A., Boyland E. (2021). Promoting Fruit and Vegetable Consumption for Childhood Obesity Prevention. Nutrients.

[54] Chen S., Binns C.W., Zhang Y. (2012). The importance of definition in diagnosing obesity: A review of studies of children in China. Asia Pac. J. Public Health.

